# Beyond CLIP: advances and opportunities to measure RBP–RNA and RNA–RNA interactions

**DOI:** 10.1093/nar/gkz295

**Published:** 2019-05-11

**Authors:** Chenyu Lin, Wayne O Miles

**Affiliations:** 1 Department of Molecular Genetics, The Ohio State University, Columbus, OH 43210, USA; 2 The Ohio State University Comprehensive Cancer Center, Ohio State University, Columbus, OH 43210, USA; 3 Center for RNA Biology, Ohio State University, Columbus, OH 43210, USA

## Abstract

RNA is an essential player in almost all biological processes, and has an ever-growing number of roles in regulating cellular growth and organization. RNA functions extend far beyond just coding for proteins and RNA has been shown to function in signaling events, chromatin organization and transcriptional regulation. Dissecting how the complex network of RNA-binding proteins (RBPs) and regulatory RNAs interact with their substrates within the cell is a real, but exciting, challenge for the RNA community. Investigating these biological questions has fueled the development of new quantitative technologies to measure how RNA and RBPs interact both locally and on a global scale. In this review, we provide an assessment of available approaches to enable researchers to select the protocol most applicable for their experimental question.

## INTRODUCTION

RNA-binding proteins (RBPs) have important functions in controlling the fate of RNAs including the modulation of pre-mRNA splicing, RNA modification, translation, stability and localization. RBPs are a diverse group of proteins that interact with RNA using an array of strategies from well-defined RNA-binding domains to disordered regions that recognize RNA sequence and/or secondary structures. This fluidity in RBP structure and target identification has made the *in silico* prediction of RBP–RNA interactions difficult. To identify RBP–RNA interactions *in vivo*, a number of experimental strategies are available. One of the most widely used approaches for detecting direct RNA–protein interactions, crosslinking and immunoprecipitation (CLIP), utilizes ultraviolet (UV) light to induce zero-length covalent bonds between RNA and the directly attached protein. Specific antibodies against the RBP of interest are then used to immunoprecipitate the RBP–RNA complexes. The purified RNA fragments can then be used to identify the position of the direct RBP–RNA interaction (1). Many of these RBP–RNA interactions are regulated by site availability and RNA structure. This has made determining the capacity of RNA to bind to other nucleotides an important and ever-expanding area of research. Emerging *in vivo* experimental tools are now available for measuring RNA structure and features. Accurately determining how different RNAs and RBPs interact and the rules that govern these decisions is key for understanding different biological processes. In this review, we first provide an overview of experimental platforms available for investigating RNA–RBP and RNA–RNA interactions and then, discuss some of the challenges that remain and outline possible solutions.

## RBP–RNA INTERACTIONS

### CLIP-seq technologies

**Figure 1.**
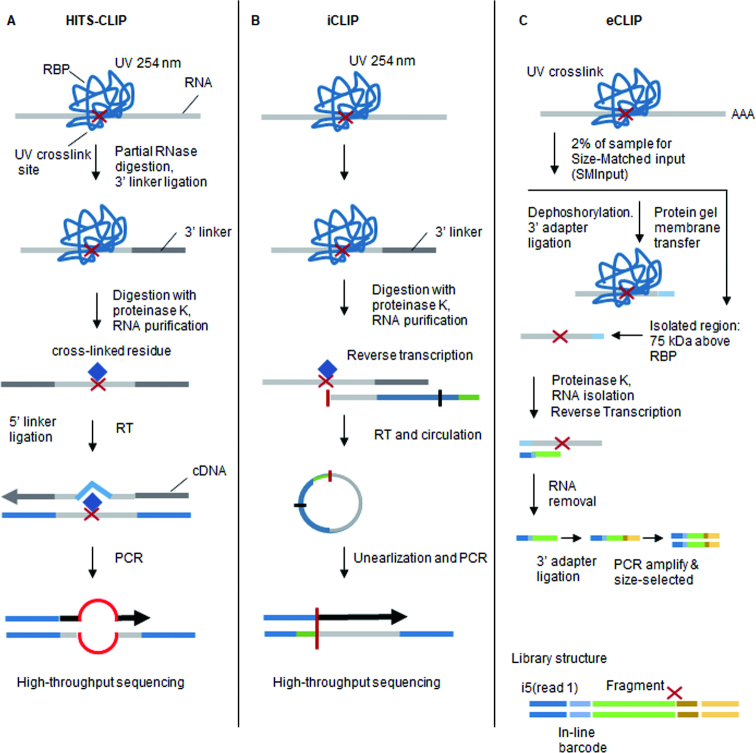
CLIP variants for studying RBP–RNA interactions (i). (**A**) High-throughput sequencing of RNA isolated by UV crosslinking and immunoprecipitation (*HITS-CLIP*) uses ultraviolet light (at the wavelength of 254 nm) to induce the formation of covalent crosslinks. The protein–RNA complexes are then immunoprecipitated using a RBP specific antibody and bound RNA measured using RNA-sequencing. (**B**) Individual nucleotide resolution CLIP (*iCLIP*) utilizes cDNA termination caused at the site of crosslinking to identify the position of RBP–RNA interaction. (**C**) Enhanced CLIP (*eCLIP*) protocol, a single adaptor is ligated at the 3′ end of the crosslinked RNA fragments, and then a second adaptor is ligated to the 3′ end of the cDNA after RT. PCR amplifies both truncated and read-through reads.

**Figure 2.**
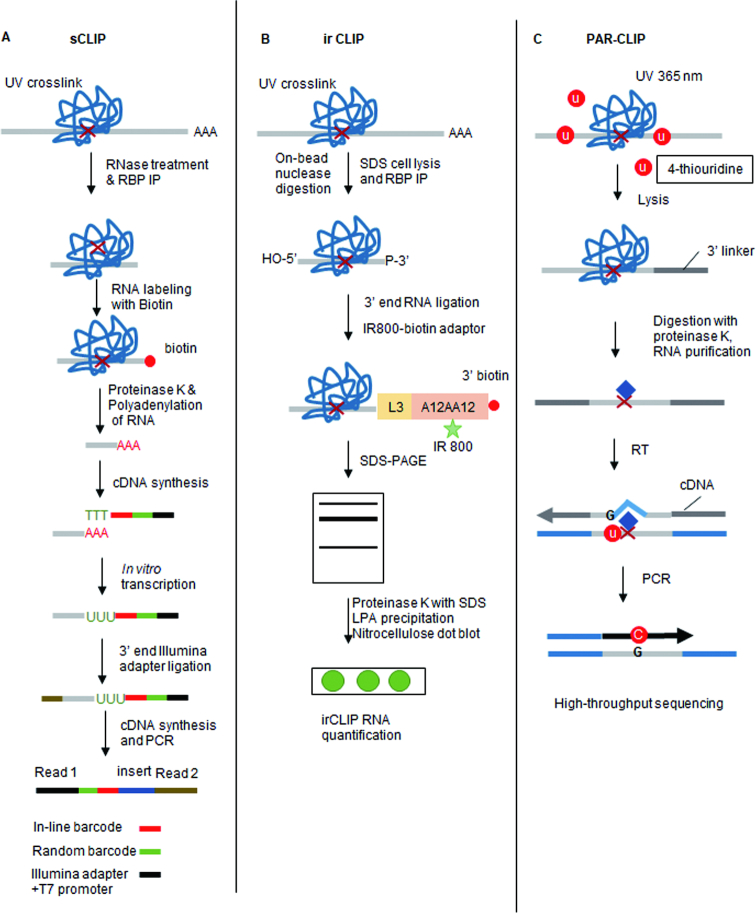
CLIP variants for studying RBP-RNA interactions (ii). (**A**) Simplified CLIP (*sCLIP)*: following immunoprecipitation of the ribonucleoprotein (RNP) complexes, the purified RNA is then poly-adenylated RNA using a modified oligo d(T) primer. (**B**) Infrared CLIP (*irCLIP*) utilizes an infrared-dye-conjugated and biotinylated ligation adaptor for rapid and quantitative analysis of *in vivo* capture protein–RNA interactions. (**C**) Photoactivatable ribonucleoside-enhanced CLIP (*PAR-CLIP*) first treats cells with 4-thiouridine (4-SU) before crosslinking cells with ultraviolet light of 365 nm and a standard CLIP protocol.

Evolution of the CLIP protocol has generated a number of variants that have improved the efficiency of multiple steps including RNA fragmentation, RBP purification and cDNA library preparation. The addition of high-throughput sequencing (HITS-CLIP) to the CLIP protocol has enabled a genome-wide view of RBP–RNA interactions and nucleotide resolution (Figure 1A). One of these derivative protocols, individual-nucleotide CLIP (iCLIP) takes advantage of residual amino acids left after the RBP–RNA crosslinking reaction to halt reverse transcriptase. By annealing a second adaptor that contains a random sequencing barcode during circularization, iCLIP enables the position of RBP–RNA interaction to be precisely mapped (2) (Figure 1B). Enhanced CLIP (eCLIP) builds on the iCLIP protocol but includes a molecular weight size-matched control during protein–RNA purification. This step provides a more accurate assessment of non-specific RNA interactions (3) (Figure 1C). Simplified CLIP (sCLIP) incorporates a poly-A polymerase step into the protocol to directly ‘polyadenylate’ all of the purified RNA fragments at the site of RBP binding (i.e. the point where reverse transcriptase stops) (4) (Figure 2A). Each of these protocols requires the use of radioactive probes that enables the excision and purification of RBP attached RNAs. To circumvent the need for radioactivity, infrared CLIP (irCLIP) uses an infrared labeled adapter to isolate RNA fragments from CLIP experiments (5) (Figure 2B). Photoactivatable ribonucleotide-enhanced CLIP (PAR-CLIP) requires the introduction of photoactivatable ribonucleotides 4-thiouridine (4-SU) or 6-thioguanosine (6-SG) into nascent RNA transcripts (6). These bases are then crosslinked to attached RBPs using 365 nm UV-A light. This crosslinking reaction frequently introduces a base transition (T-C for 4-SU or G-A for 6-SG) at the RBP-binding site (Figure 2C). This enables the position of RBP–RNA interaction to be mapped using RNA-seq.

These protocols have formed the foundation for additional CLIP strategies that are designed to purify specialized RBP–RNA complexes. These include the miRNA machinery (Ago2 HITS-CLP) (7) and RNA methylation marks (m5C-miCLIP and m6-miCLIP) (8). CLIP methods that detect rare RNA methylation events have been particularly successful and crosslink either an enzymatically inactive RNA methylase (NSUN2) (m5C-miCLIP) (8) or an antibody (m6-miCLIP) (9) to sites of RNA methylation.

One of the long-standing issues with CLIP approaches has been the high level of non-specific RNA that is purified. In an attempt to minimize this issue, a number of groups have shifted to using epitopes tags and denaturing wash conditions. The crosslinking and analysis of cDNAs (CRAC) protocol employs a two-step affinity purification of tagged proteins under denaturing conditions (10). More recently, several additional denaturing CLIP protocols have become available, including the sequential histidine- and streptavidin-based affinity purification (CLAP), urea-iCLIP (uvCLAP) and denaturing CLIP (dCLIP) (11–15). The specificity gained by purifying RBP–RNA complexes under denaturing conditions reduces the need for further purification using gel electrophoresis.

#### Analysis and normalization of CLIP-seq data

All polymerase chain reaction (PCR)-based library preparations have the potential to contain products generated by PCR and primer errors. To account for this during CLIP library construction, the use of unique molecular identifiers (UMIs) within the reverse transcription primer has become standard. This makes it possible to identify the effects of PCR amplification biases (16). Several computational tools including iCount, PARalyzer and wavClusteR enable the accurate quantitation of CLIP RNA-Seq data (17). These programs separate somatic variants from CLIP signal and reduce the background generated by genetic variation. Once the CLIP data is aligned, motif finding tools, such as DREME and HOMER, can be used to predict the motifs bound by RBPs and to generate position weight matrices (18,19). Newer tools, including Zagros, combine secondary structure and seed sequence information to enhance motif calling (20). The kpLogo tool takes advantage of the relatively short motifs bound by RBPs, compared to their DNA binding counterparts, to predict interaction sites (21). The beRBP program uses a combination of RBP nucleotide preference and RNA sequence to build position weight matrices of putative RBP interactions (22). Collectively, the addition of UMIs and powerful mapping and RBP interaction site prediction tools have improved the CLIP protocol.

The standardization of the CLIP approach has facilated an ever-growing number of RBP–RNA interactomes to be identified. The availability of these datasets has enabled the ENCODE consortium to integrate RBP–RNA maps to generate predictive regulatory networks of RBP and RNA interactions. Using this resource, new pre-published work has used 233 eCLIP datasets to construct these networks. They then depleted (shRNA) or deleted (CRISPR) RBPs and used RNA Bind-N-seq and CLIP-seq platforms to map RBP-controlled biological processes. This unique approach builds on existing datasets and has expanded the catalog of functional elements encoded in the human genome (23).

#### Limitations of CLIP protocols

These protocols enable the identification of regions of RBP–RNA interaction across different cell types. Although these approaches continue to improve our understanding of RBP-binding sites, they do have numerous shortcomings that should be considered. PAR-CLIP based protocols are currently limited to cell culture based models and cannot be used for tissue studies. This is due to toxicity issues associated with 4-SU or 6-SG treatment. Some of these protocols are also technically challenging, for example, the iCLIP approach is prone to high experimental failure rates that result in low RNA purification levels. More generally, three significant limitations should be considered when designing and conducting CLIP-based assays. First, RBP binding does not itself imply regulation, as interactions between an RBP and an RNA can be transient or occur via cooperative binding events. Second, these assays only enable an ‘average’ of RBP binding across an often heterogeneous population of cells. This approach cannot account for transcriptional or post-transcriptional differences between cells within a population. Finally, kinetic measurements of RBP–RNA interactions via CLIP pipeline are difficult due to inefficient crosslinking.

### Other methods to investigate RBP–RNA interactions

**Figure 3.**
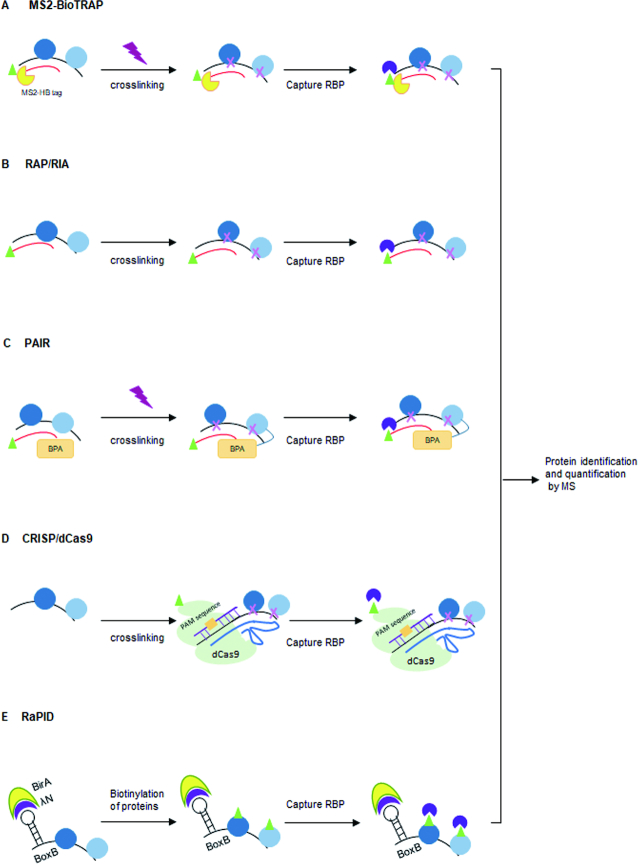
Non-CLIP based protocols for capturing RNA and RBP interactions. After crosslinking, target mRNP complexes can be specifically captured using: (**A**) an MS2 *in vivo* biotin-tagged RNA affinity purification; (**B**) biotinylated anti-sense RNA oligonucleotides and streptavidin beads; (**C**) Peptide nucleic acid bases on a covalent crosslink between the photoactivatable amino acid adduct *p*-benzophenylalanine (BPA) and the nearest RBP; (**D**) the CRISPR*/*dCas9 system. (**E**) RNA–protein interaction detection (RaPID) technology uses a biotin ligase domain (λN-HA-BirA*) to biotinylate proteins that bind BoxB sites (blue) within a RNA sequence of interest.

RNA affinity tags enable the co-purification of labeled RNAs and their protein cargos. In these experiments, cells are transfected with plasmids expressing an aptamer-tagged RNA of interest. These modified RNA hybrids can be adapted to directly address specific experimental questions. RNA affinity in tandem (RAT) uses two affinity tags, PP7 and tobramycin, to capture RNA–RBP interactions under stringent conditions (24). RBP purification and identification (RaPID) use an MS2 stem–loop RNA to specifically capture the MS2 coat protein fused with a fluorescent label and a Streptavidin-binding motif. This allows the cellular location of the protein to be identified and permits the purification of RBP–RNA complexes via Streptavidin pulldown. This protocol can also be adapted to biotinylate RBPs that interact with the MS2 stem–loop containing RNAs that also express the MS2 coat protein fused to a biotinylation domain (MS2-BioTRAP). The proteins can then be purified and identified using Mass Spectrometry (25) (Figure 3A).

The limitation of aptamer tagging of target RNA is that these techniques require the production of an exogenous RNA or RBP that may be prone to aggregation and/or mis-localization. Emerging nucleic acid hybridization-related strategies that circumvent some of these potential issues are now available including RNA antisense purification sequencers (RAP RNA-seq) (26) and RNA interactome analysis with new generation sequencers (RIA-seq) (27). These protocols use biotinylated antisense probes to capture endogenous RNAs and their protein cargos (Figure 3B). Peptide nucleic acid (PNA)–RNA based approaches utilize a cell penetrating peptide to introduce a biotinylated oligomer that binds to complementary sequences within its endogenous target RNA (28). Following UV crosslinking, the RBP–RNA complexes can be isolated using streptavidin to purify the biotinylated PNA oligomer and the RNA–RBP cargo (Figure 3C).

An adapted CRISPR/dCas9 system can also be used to capture RNA–chromatin interactions (29) and track endogenous RNA–RBP complexes (30). This system comprises: (i) a biotinylated dCas9 (29–32), (ii) a guide RNA (sgRNA) that matches the target ssRNA and (iii) a short PAM presenting DNA oligonucleotide (PAMmer). The oligonucleotide works *in trans* and binds upstream of the target ssRNA sequence. This allows the sgRNA to recognize the specific RNA target instead of the encoding DNA locus (Figure 3D). One likely future improvement of this technology may come from using the Cas13 enzymes that have nucleotide-binding (HEPN) endoRNase domains, as these may provide a simpler strategy for purifying RNA than dCas9 (33).

Many of these technologies do show some tendency for detecting proteins that interact post-lysis (34–38). The RNA–protein interaction detection (RaPID) method overcomes this issue by using a modified promiscuous biotin ligase protein domain, BirA*, fused to an RNA-binding domain. This fusion protein biotinylates proteins that interact with the introduced RNA motifs and then uses streptavidin purification and mass spectrometry for their identification (39) (Figure 3E).

## INTERACTION BETWEEN RNAs AND RNAs/RBP

### Identification of RNA–RNA interactions mediated by RBPs

**Figure 4.**
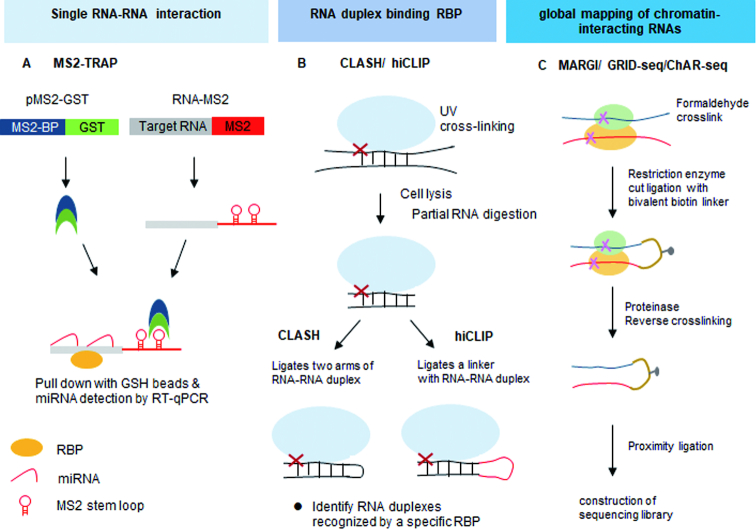
High-throughput approaches for studying RNA–RNA interactions. (**A**) MS-tagged RNA affinity purification (*MS2-TRAP*) requires cells to express the pMS2-GST fusion protein and pMS2-target RNA. RNP complexes are then affinity-purified using GSH beads. (**B**) The crosslinking, ligation and sequencing of hybrids *(CLASH)* protocol use chimeric reads in RNA-Seq data following RNA–protein capture to computationally determine position. Hybrid and individual nucleotide resolution ultraviolet crosslinking and immunoprecipitation (*hiCLIP)* coupled with the CLASH protocol introduces a linker between the two arms of an RNA duplex. (**C**) Mapping RNA–genome interactions (*MARGI)* utilizes formaldehyde to crosslink protein–RNA–DNA complexes. These fragments are then ligated to a biotinylated half-RNA-half-DNA linker via proximity ligation. The resulting chimeric RNA–DNA sequences can then be Streptavidin purified. Similar to MARGI, the difference procedures of *ChAR-seq* and *GRID-Seq* use intact nuclei, restriction enzyme digestion, RNA-linker ligation and proximity ligation.

An increasing number of non-canonical miRNA-binding modes have recently been described that make predicting miRNA binding solely based on the canonical miRNA seed site insufficient. miRNAs can bind in a number of distinct ways that include supplementary pairing of miRNA 3′-bases, 3′-end centric ‘seedless’ pairing, center miRNA pairing and nucleation bulges in the seed region (40–42). To investigate miRNA binding *in vivo*, emerging approaches are available that can detect many of these non-canonical interactions. One strategy to identify these interactions is via MS2-tagged RNA affinity purification protocol (discussed above) (MS2-TRAP) (Figure 4A). This experimental strategy uses RNA-tags to purify miRNA-Argonaute 2 (Ago2) complexes, on substrates of interest. Ago2 HITS-CLIP is a more commonly used approach to identify Ago2-bound miRNA target sites and uses the standard CLIP protocol to determine RNAs bound by miRNA-loaded Ago2 (7). A modified AGO2 HIST-CLIP protocol referred to as CLEAR (covalent ligation of endogenous Argonaute-bound RNAs-CLIP) uses an additional ligation reaction to link the miRNA and its endogenous mRNA target together. This has been shown to enrich for Ago2 bound miRNAs/mRNAs complexes (43).

Local RNA secondary structure has an important role in regulating the capacity of RBPs to interact with their RNA substrates. Crosslinking, ligation and sequencing of hybrids (CLASH) was one of the initial tools that enabled the identification of *in vivo* RNA duplexes (Figure 4B) (44). CLASH is a modification of the traditional CLIP protocol, but includes an additional proximity ligation step. This ligates each arm of the RNA duplex together to allow RNA–RNA interactions mediated by an RBP to be identified. There are several limitations in the CLASH protocol that should be considered including: the ligation the two arms of RNA duplexes requires a region of at least an eight single-stranded nucleotides for efficient circularization (45,46) and separating the individual arms of hybrid RNAs after direct ligation can be computationally challenging (47). To overcome these issues, RNA hybrid and individual-nucleotide resolution CLIP (hiCLIP) introduces a unique ligation step that incorporates a linker between each of the ligated arms of the RNA duplex (48) (Figure 4B). The addition of this linker significantly improves the efficiency of the ligation reaction, and it also provides an additional quality control step as all hybrid reads arise from a controlled ligation reaction that enables the identification of each arm within the hybrid (48).

Noncoding RNAs (ncRNAs) have a remarkable number of biological functions. NcRNAs operate as RNA–protein complexes and also act on other nucleic acids that utilize base pairing interactions to selectively bind. Identifying the proteins and nucleic acids that interact with ncRNAs is likely to further expand our understanding of their function. To detect LNC–RNA–chromatin interactions, multiple methods are available. These include capture hybridization analysis of RNA targets (CHART) (49), chromatin isolation by RNA purification (ChIRP) (50), RNA antisense purification sequencers (RAP RNA-seq) (51) and RNA interactome analysis with new generation sequencers (RIA-seq) (27). These protocols use anti-sense biotinylated probes to ‘capture’ a specific RNA and its cargo via Streptavidin purification.

### Genome-wide identification of RNA–RNA interactions and RNA structure

**Figure 5.**
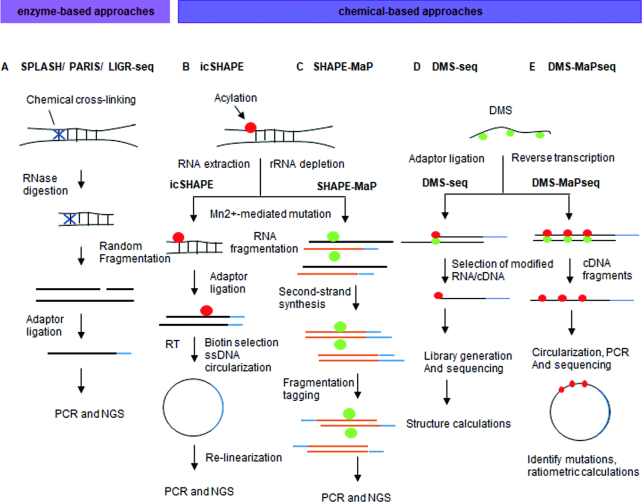
Chemical-based approaches for studying RNA–RNA interactions. (**A**) Psoralen-based crosslinking methods, including selected hybrids (SPLASH), psoralen analysis of RNA interactions and structures (PARIS), and ligation of interacting RNA followed by high-throughput sequencing (LIGR-seq), rely on psoralen to induce crosslinks between double-stranded uridines followed by UV irradiation. (**B**) *In vivo* click selective 2-hydroxyl acylation and profiling experiment (icSHAPE) treats cells with NAI-azide, allowing for attaching a biotin moiety through a CLICK reaction. SHAPE-treated RNA fragments are then purified by streptavidin and then converted into a sequencing library. (**C**) SHAPE and mutational profiling (SHAPE-MaP) uses 2′-hydroxyl-selective reagents that react to form covalent 2′-O-adducts at conformationally flexible RNA nucleotides, which will be misread during reverse transcriptase. The positions and frequencies of SHAPE adducts are recorded as mutations in the cDNA primary sequence. (**D**) Dimethyl sulfate (DMS) specifically modifies unpaired adenines and cytosine’s *in vivo* at their Watson–Crick base-pairing positions. This modification terminates the reverse transcriptase allowing analysis of RNA structure on a global scale. (**E**) Dimethyl sulfate mutational profiling with sequencing (DMS-MaP-Seq) uses DMS and a thermostable group II intron reverse transcriptase (TGIRT) for both genome-wide studies and focused *in vivo* investigations of even low abundance RNAs.

The CLASH protocol (see RNA–RNA interactions mediated by a specific RBP section above) can identify *in vivo* RNA duplexes recognized by RBPs in a high-throughput manner. It is however limited in its capacity to determine the structure of unbound RNA regions. To globally map RNA structures, several protocols leverage enzymes or chemicals that specifically react with local RNA features and/or structures. Enzyme-based methods include: sequencing of psoralen-cross-linked, ligated and selected hybrids (SPLASH) (Figure 5A) (52), psoralen analysis of RNA interactions and structures (PARIS) (Figure 5A) (53), and ligation of interacting RNA followed by high-throughput sequencing (LIGR-seq) (Figure 5A) (54). These approaches use proximity ligation to produce chimeric sequences between interacting RNA–RNA complexes. These RNA–RNA duplexes are then crosslinked using inter-collating psoralen-based chemicals and the hybrids fragmented and converted into a RNA sequencing library. The resulting mixture of RNA fragments can then be used to assess nucleotide accessibility and enables the identification of RNA–RNA interactions and secondary structure.

Alternative experimental strategies use chemical-based approaches to map RNA structure. These protocols include selective 2′-hydroxyl acylation analyzed by primer extension (SHAPE) (55) (Figure 5B), selective 2-hydroxyl acylation analyzed by primer extension and mutational profiling (SHAPE-MaP) (56) (Figure 5C), dimethyl sulfate sequencing (DMS-Seq) (57) (Figure 5D), high-throughput sequencing for chemical probing of RNA structure (Mod-seq) (58) and structure-seq (59), chemical inference of RNA structure sequencing (CIRS-seq) (60). These technologies utilize membrane permeable chemicals to assess RNA structure. Such approaches use a number of different experimental strategies to identify RNA structure including nucleobase-specific chemicals, carbodiimide modifying reagents and ribose-specific probes. In dimethyl sulfate (DMS)-based approaches, DMS is used to alkylate the base-pairing faces of unpaired adenosine and cytidine nucleotides. These marks can then be utilized to identify the structure of the RNA and the attached RNA–protein complexes (RNPs) (61). However, DMS-based protocols have traditionally found detecting RNA of low abundance difficult. To overcome this issue, dimethyl sulfate mutational profiling with sequencing (DMS-MaP-Seq) (Figure 5E) uses DMS-induced lesions to generate mutations rather than cDNA truncations through the use of the thermostable group II intron reverse transcriptase (TGIRT). This makes it possible to detect low abundance RNAs and to identify the structured regions of these RNAs (62). SHAPE-related protocols use hydroxyl-selective electrophiles to modify the 2′-hydroxyl groups of unbound single-stranded nucleotides. One advantage of SHAPE over nucleobase-specific probes is its capacity to modify all four nucleotides rather than a specific subset. Future protocols are likely to integrate multiple chemical and enzymatic steps into RNA structure experiments to unveil a more comprehensive picture of RNA folding.

### Global analysis of effect of RNA secondary structure on RNA–RBP interaction

There are significant discrepancies between the prediction of RNA-binding events and the number of these sites that are occupied within cells. RNA secondary structure is a strong determinant that can either facilitate or inhibit RBP binding. To address the role of RNA structure in controlling RBP–RNA interactions, multiple high-throughput technologies have been designed. One of these protocols, RNAcompete, is a high-throughput *in vitro* binding assay that identifies RBP-binding motifs by quantifying the relative affinity of an RBP for a pre-defined set of RNAs (63). To map the contribution of RNA secondary structure in regulating RBP interaction, RNA Bin-n-Seq (RBNS) (64) and RNAcompeteS (65) use epitope tagged protein to purify target RNA from pools of random RNA sequences flanked by sequencing adapters.

**Figure 6.**
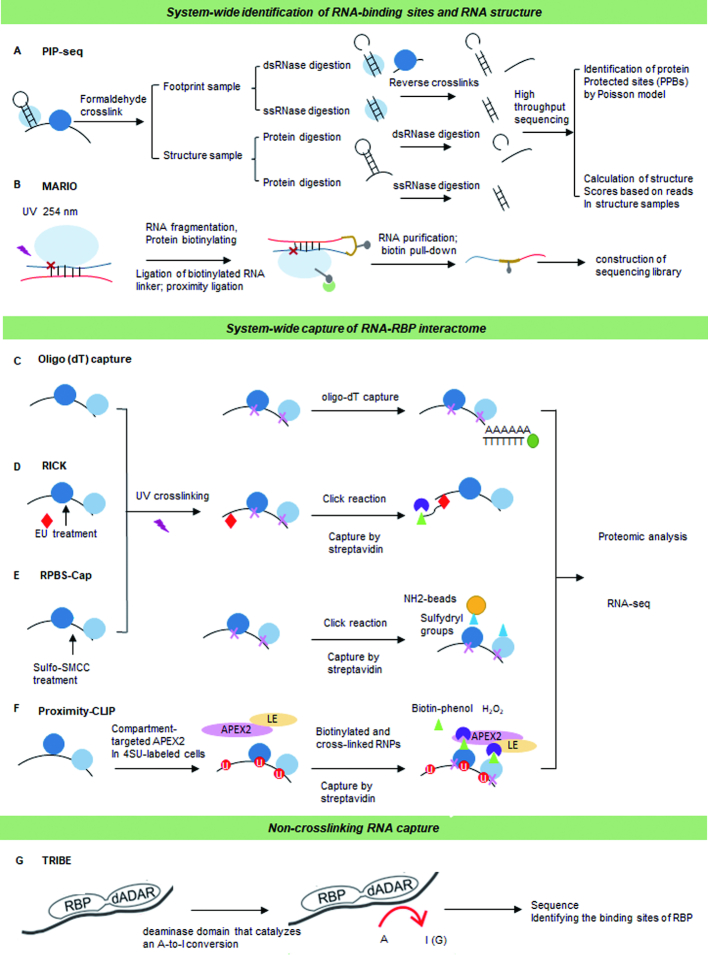
System-wide identification of RNA interaction. (**A**) Protein interaction profiling-sequencing (*PIP-seq*) crosslinks sites of RNA–protein interaction followed by subsequent RNase digestion of ssRNA and dsRNA regions before high-throughput sequencing. (**B**) Mapping RNA interactome *in vivo* (*MARIO)* requires RNA and RBP crosslinking before RNA fragmentation and biotinylation of the RBPs. This allows the RBP to immobilized and proximal RNA molecules to be ligated with a biotinylated RNA linker to form a chimeric RNA. (**C**) *mRNA interactome capture* allows identification of RBPs that associate with mRNAs in cells. This approach employs UV crosslinking that covalently links RNAs with interacting RBPs and then covalently bound RBPs are isolated using oligo (dT) magnetic beads. (**D**) RNA and click chemistry (*RICK*) biotinylates new synthesized RNAs using EdU incorporated residues. Streptavidin is then used to capture the protein complexes bound. (**E**) Capture of protein-binding sites on RNAs assay (RPBS-Cap) utilizes the Sulfo-SMCC chemical to covalently conjugate the proteins of within RNP complexes to amine-containing beads. (**F**) Proximity-CLIP utilizes an APEX2 fusion protein to add biotin-phenol groups to proteins within a specific cellular compartment. Cells then grown in 4SU to label the RNA before APEX2 are activated resulting in the APEX-mediated BP oxidation, and a PAR-CLIP-like protocol is used to capture the RBP–RNAs by streptavidin affinity chromatography. (**G**) Targets of RNA-binding proteins identified by editing (*TRIBE)* fuses the deaminase domain of the ADAR protein to a RBP of interest. The editing specificity of the fusion protein is able to catalyze an adenosine-to-inosine conversion allowing the RNA recognition features of the RBP to be identified.

Each approach yields a comprehensive profile of the sequence and RNA secondary structural preferred by candidate RBPs. Data from these pipelines have revealed that many RBP-binding sites have low compositional complexity, but have extensive preferences for contextual features. These extend beyond the traditional short linear RNA motifs to include RNA secondary structure, flanking nucleotide composition and bipartite motifs (66). Protein interaction profile sequencing (PIP-seq) utilizes formaldehyde to crosslink RNA–protein interactions. The RNA is then digested with structure-specific RNases to leave single stranded RNA (ssRNA) and double stranded RNA (dsRNA) fragments that are used for RNA-Seq (67) (Figure 6A). This approach has identified putative binding motifs for numerous RBPs and has provided novel insights into the cooperative binding by RBPs (67). Mapping RNA interactome *in vivo* (MARIO) is an alternative method that enables the identification of protein-assisted inter- and intra-molecular RNA interactions as well as RNA structures (68) (Figure 6B). MARIO leverages a biotinylated RNA linker to form a chimeric RNA that can be isolated and sequenced (68). In contrast to psoralen-based approaches, MARIO identifies all RNA pairs bound to RBPs without requiring RNA–RNA hybridization. In the future, the integration of MARIO with psoralen-based methods may provide a more comprehensive view of the RNA interactome.

## LIMITATIONS AND FUTURE DIRECTIONS

In this review, we have discussed protocols that enable the identification of RNA–protein and RNA–RNA interactions. However, as the number of different experimental technologies grows, so do the technological challenges. Here, we discuss areas of opportunity for improving the detection of RBP–RNA interactions and new RNA capture techniques.

### Improving capture RNAs

Refining the signal-to-noise ratios for existing oligo-dT capture protocols is a significant challenge. The use of locked nucleic acids (LNA) rather than oligo-dT to purify polyadenylated RNA may provide the solution for this issue (69) (Figure 6C). LNAs have a higher melting temperatures than deoxy (dT)s, and so LNA-based capture allows for higher stringency washing steps during RNA purification (70). These experimental modifications have improved the mRNA capture pipeline; however, they are also costly and may currently be beyond the fiscal reach of many labs. One alternative may be the use of silica instead of oligo (dT). This method named 2C utilizes silica columns to bind and purify nucleic acids along with their covalently crosslinked protein cargos (71). This method bypasses the need for immunoprecipitation and radiolabeling, making it cheaper and more straightforward compared with other capture protocols.

### Global capture RNA interactome

Many technologies investigate interacting partners of one RBP at a time. This, however, limits our ability to study the global landscape of RNPs and to determine the combinatorial binding and regulation of RBPs. Recent advanced methods now enable the global mapping of RBP–RNA interactions, including global PAR-CLIP (gPAR-CLIP) and protein occupancy profiling (72,73). These technologies are based on the capture of polyadenylated RNAs using oligo (dT). To systemically capture proteins bound to newly synthesized or non-polyadenylated RNAs, RNA interactome using click chemistry (RICK) utilizes 5-ethyluridine (5-EDU). Cells are treated with 5-EDU and it becomes incorporated into newly synthesized RNA. The 5-EDU is then biotinylated using a click reaction and the 5-EDU labeled RNA–protein interactome purified using streptavidin (Figure 6D) (74).

### Capturing RBPs and their RNA cargo without antibodies

The RPBS-Cap capture protein and binding RNA protocol leverages a chemical reagent, Sulfo-SMCC (sulfosuccinimidyl 4-[N-maleimidomethyl] cyclohexane-1-carboxylate), rather than an antibody to covalently conjugate the amine groups within the RBPs into protein G beads (75) (Figure 6E). This is then used to purify the RBP–RNA complexes. The cellular localization of RNA has an important regulatory role in modulating gene expression. To study how different cellular localizations of RNA modify the interaction with RBPs, proximity-CLIP was designed. This protocol uses APEX2-mediated proximity biotinylation of proteins coupled with PAR-CLIP. Alterations within the APEX2-protein via the addition of nuclear or cytoplasmic signals enable the RNA to be transported to different regions within the cell and RBP–RNA capture is achieved via PAR-CLIP based methods (76).

### Improving crosslinking

One of the benefits of UV crosslinking is that it requires direct contact between an amino acid and a nucleotide for the reaction. There are however some limitations in this process including UV-crosslinking has low efficiency (0.1–5%), a bias in the crosslinking levels of different RBPs at varying wavelengths and difficulty in crosslinking RBPs that interact with double-stranded RNA (77). One potential strategy to improve UV-crosslinking may be UV pulsed lasers (78). These lasers emit 266 nm UV radiation at >106 W/cm^2^ on a nanosecond timescale, and this approach may significantly increase crosslinking efficiencies to levels approaching 50% (79). Preliminary studies have found that this technology can improve crosslinking efficiency between protein and DNA and suggests that UV lasers may also produce improved RNA/RBP crosslinking efficiency for CLIP. The major hurdle with this protocol is the limited availability of the laser and their trained operators and the overall high cost. However, given that RBPs dynamically interact with RNA, this nanosecond-based crosslinking may provide a novel method for detecting the number of proteins bound to a single RNA molecule, and global RNA-binding interaction rates.

Poor UV-crosslinking efficiency is a problem for capturing RBPs that interact with dsRNA. One potential reagent that may circumvent many of the technical issues of RBP–dsRNA capture is methylene blue (80). Methylene blue intercalates between the bases of dsRNA, and ‘opens up’ the RNA structure to permit crosslinking of dsRNA to RBPs in the presence of visible light (80). Additionally, methylene blue may also be integrated into 254 nm UV crosslinking reactions to aid in the capture of both single and double-stranded RNA interactions.

### Non-crosslinking alternatives

Low UV cross-linking efficiencies and sequence biases have led to the development of experimental strategies that ‘label’ the RNA rather than purify the RBP and its substrates. The target of RNA-binding proteins identified by editing (TRIBE) approach is based on a fusion protein consisting of the deaminase domain from the ADAR family of RNA-editing enzymes and an RBP that marks target RNAs near the RBP-binding sites by an A-to-I change (81). The advantage of this method is that it eliminates the need for crosslinking and immunoprecipitation steps (Figure 6G). However, low editing efficiency and sequence bias of the ADARcd domain have made identifying substrates of some RBPs difficult. The recent advent of Hyper TRIBE that incorporates a hyperactive mutation, E488Q, into the ADARcd domain has overcome the majority of these issues (82) though some selection and efficiency biases do remain. Additional protocols using homologous chimeric approaches have fused RNA-binding proteins of interest with a poly-(U) polymerase (PUP). This chimeric protein leads to the addition of a string of uridine base-pairs (the ‘U-tag’) specifically in the RNA bound by the RBP–PUP fusion. These U-tagged RNAs can then be identified by RNA-Seq (83).

### Perspective

This is an exciting time to be investigating the roles of RNA and their complex relationships with RBPs. An evergrowing body of work has found important functions for these interactions in almost every biological system. The protocols and technological advances/possibilities discussed within this review will continue to grow, expanding our capacity to quantitate how different RNA species fold, function and complex with RBPs in different cellular conditions.
